# Cardiovascular Responses Induced by Obstructive Apnea Are Enhanced in Hypertensive Rats Due to Enhanced Chemoreceptor Responsivity

**DOI:** 10.1371/journal.pone.0086868

**Published:** 2014-01-23

**Authors:** Juliana M. M. Angheben, Guus H. M. Schoorlemmer, Marcio V. Rossi, Thiago A. Silva, Sergio L. Cravo

**Affiliations:** Department of Physiology, Escola Paulista de Medicina, Federal University of São Paulo, São Paulo, Brazil; King’s College London School of Medicine, United Kingdom

## Abstract

Spontaneously hypertensive rats (SHR), like patients with sleep apnea, have hypertension, increased sympathetic activity, and increased chemoreceptor drive. We investigated the role of carotid chemoreceptors in cardiovascular responses induced by obstructive apnea in awake SHR. A tracheal balloon and vascular cannulas were implanted, and a week later, apneas of 15 s each were induced. The effects of apnea were more pronounced in SHR than in control rats (Wistar Kyoto; WKY). Blood pressure increased by 57±3 mmHg during apnea in SHR and by 28±3 mmHg in WKY (p<0.05, n = 14/13). The respiratory effort increased by 53±6 mmHg in SHR and by 34±5 mmHg in WKY. The heart rate fell by 209±19 bpm in SHR and by 155±16 bpm in WKY. The carotid chemoreceptors were then inactivated by the ligation of the carotid body artery, and apneas were induced two days later. The inactivation of chemoreceptors reduced the responses to apnea and abolished the difference between SHR and controls. The apnea-induced hypertension was 11±4 mmHg in SHR and 8±4 mmHg in WKY. The respiratory effort was 15±2 mmHg in SHR and 15±2 mmHg in WKY. The heart rate fell 63±18 bpm in SHR and 52±14 bpm in WKY. Similarly, when the chemoreceptors were unloaded by the administration of 100% oxygen, the responses to apnea were reduced. In conclusion, arterial chemoreceptors contribute to the responses induced by apnea in both strains, but they are more important in SHR and account for the exaggerated responses of this strain to apnea.

## Introduction

Several studies suggest that overactive carotid chemoreceptors can lead to sympathetic activation and hypertension. This mechanism may contribute to hypertension induced by obstructive sleep apnea [1], [2], [3] and by other causes [4], [5], [6], [7], [8]. Chemoreceptor inactivation has therefore been proposed as a treatment for hypertension [9].

Spontaneously hypertensive rats are commonly used to study mechanisms of hypertension because of their similarities with hypertensive human patients, including sympathetic activation and chemoreceptor function. Several studies have shown that carotid chemoreceptors are larger and more sensitive in the spontaneously hypertensive rat (SHR) than in normotensive controls [10], [11], [12]. It has been suggested that increased carotid chemoreceptor activity contributes to the development and maintenance of hypertension in the SHR [13], [14], presumably through chemoreceptor-related stimulation of sympathetic activity.

Although evidence that afferent activity of the carotid body is increased in SHR is strong [10], [15], [12], [11], evidence that acute respiratory and cardiovascular responses to chemoreceptor stimulation are increased in SHR is not. Several studies done in anesthetized rats failed to find convincing proof (see references in [16]). In awake SHR, acute chemoreceptor activation by hypoxia caused changes in heart rate and arterial pressure similar to those seen in normotensive rats, but caused a larger changes in breathing rate and in indicators of vagal and sympathetic activity [16], [17]. Cardiovascular responses in SHR to acute chemoreceptor stimulation with cyanide may be either increased [14] or normal [18].

Recently we developed a tracheal balloon to induce obstructive apnea in unanesthetized rats [19]. The balloon is contained in a rigid plastic tube, which permits induction of apnea without tracheal pain. Balloon inflation induces hypoxia, hypercapnia, and respiratory effort that are similar to those observed in humans. In the current study, we investigated the cardiovascular responses induced by obstructive apnea in hypertensive rats and the role of chemoreceptors in these responses. The role of arterial chemoreceptors in apnea-induced responses was examined in two ways: by ligation of the carotid body arteries and by increasing the oxygen content of the inspired air. We also compared apnea-induced responses in SHR to those of normotensive control rats (WKY). As anesthesia affects responses to apnea and chemoreceptor stimulation, all experiments were done in unanesthetized animals.

## Methods

### Animals and ethical approval

We used male spontaneously hypertensive rats (SHR) and Wistar Kyoto rats (WKY) weighing 270 to 310 g. Rats were housed individually in a room with controlled temperature and a 12:12 h light-dark cycle and had free access to water and food. Experiments followed the Guiding Principles in the Care and Use of Animals of the American Physiological Society and were approved by the Ethics in Research Committee of the Federal University of Sao Paulo (protocol 0165/11).

### Surgical preparation

Rats were anesthetized with ketamine (100 mg/kg body weight i.p.) and xylazine (20 mg/kg body weight i.p.) and received ketoprofen (2 mg/kg s.c.) after surgery.

*Tracheal balloon for induction of apnea*: We used polyurethane balloons contained in a thin-walled Teflon tube (6 mm long, 2.2 mm o.d.). The lumen of the Teflon tube could be closed by injecting water into the balloon and opened by withdrawing the injected water. The balloon assembly was implanted through a small T-shaped cut in the trachea. The free end of the balloon catheter was led subcutaneously to the back of the neck, and it was connected to an elbow made of 23 g hypodermic tubing that protruded through the skin. The balloon catheter was filled with saline, the connector was closed with a plastic cap, and the skin incisions were sutured closed.

To inflate the balloon, the elbow on the rat’s back was connected to a Hamilton syringe filled with water with PE50 tubing. The syringe was driven by a solenoid, as described previously [19]. Because the balloon is contained in a rigid Teflon tube, balloon inflation does not cause stimulation of tracheal pain receptors. The balloons, when not inflated, do not significantly increase resting respiratory effort.

*Balloon catheter for the measurement of intrathoracic pressure:* We used a polyurethane balloon-tipped catheter with a thin-walled, non-elastic tip of ∼4 mm in diameter [19]. The balloon catheter was filled with saline and placed on a metal guide wire. The balloon tip was placed under the sternohyoid muscle and gently advanced along the right side of the trachea to enter the mediastinum. The free end of the balloon catheter was led subcutaneously to the back of the neck and exteriorized as the tracheal balloon. To measure thoracic pressure changes, the balloon catheter was connected to a disposable pressure transducer. Thoracic pressure signals were smoothed with a 200 ms triangular window to remove heart beat artifacts.

*Arterial pressure cannula:* Arterial cannulas were made from 4 cm of PE10 tubing, 0.28 mm i.d. x 0.64 mm o.d. (BB31695-PE/1, Scientific Commodities Inc., Lake Havasu City, AZ, USA), heat-welded to PE 50 tubing. The PE10 tip was stretched in steam to ∼0.5 mm o.d. The PE10 tip was inserted in the femoral artery. The free end was led subcutaneously to the back of the neck and exteriorized as the tracheal balloon. The cannula was filled with saline containing heparin (50 U/mL) and penicillin G (1.2 mg/mL). The cannula was flushed once a week or on the day before an experiment. To measure arterial pressure changes, the catheter was connected to a disposable pressure transducer.

*Carotid artery cannula for collection of blood samples:* The left common carotid artery was cannulated with silicone-tipped PE50 tubing. The free end of the cannula was led subcutaneously to the back of the neck and connected to a 23 g stainless steel elbow. The cannula was filled with saline containing heparin (50 U/mL) and penicillin G (1.2 mg/mL), and closed with a plastic cap.

*Venous cannula for drug infusion:* Venous cannulas were made from 4 cm of silicone rubber tubing, 0.6 mm i.d., 1 mm o.d., (BB518-20, Scientific Commodities Inc., Lake Havasu City, AZ, USA) connected to PE 50 tubing. The silicone tip was inserted into the femoral vein. The free end was led subcutaneously to the back of the neck and was exteriorized as the tracheal balloon.

*Carotid chemoreceptor inactivation:* Under a surgical microscope, the carotid bifurcation was exposed, and the carotid body artery was isolated and tied [20]. The ligated carotid body artery was cut distal to the ligature, and the skin incision was closed. The procedure was done bilaterally. This method allows normal function of the carotid baroreceptors [20]. Chemoreceptor inactivation was confirmed two days later by analyzing the responses to the i.v. injection of KCN in the awake rat, as described below.

*ECG electrodes:* ECG electrodes (0.8-mm stainless steel wire loops) were implanted through the skin with a hypodermic needle used as a guide. To record the ECG, electrodes were connected to an ML 136 bio amp (ADInstruments, Melbourne, Australia).

### Design of experiments

**Cardiovascular responses to obstructive apnea.** SHR and WKY rats were fitted with tracheal balloons, arterial and venous catheters, and ECG electrodes. They were allowed to recover from surgery for at least 7 days. Rats were placed in a plastic box (volume of 6 L) that could be closed. Cannulas and electrodes were connected through a narrow slit (0.5×10 cm) in the top of the box. The cage was filled with either room air or with 100% oxygen. Starting one minute later, 5 apneas of 15 seconds each were induced, with an interval between apneas of 2 minutes. Arterial and thoracic pressure and ECG were recorded during the experiment. The O_2_ content of the box was changed, and an additional series of 5 – 10 apneas was made. The oxygen concentration in the cage was monitored with a portable oxygen monitor (OM-200, RTC Hospitalar, São Paulo, Brazil). Cyanide (KCN, 40 µg in 0.1 mL) was then injected through the venous cannula to stimulate the arterial chemoreceptors.

One day later, the rats were anesthetized, and both carotid body arteries were ligated and cut. Two days after surgery, the responses to apnea were measured again. Cyanide (KCN, 40 µg in 0.1 mL) was then injected through the venous cannula to confirm carotid chemoreceptor inactivation.

**Arterial blood gases during apnea.** SHR and WKY rats (n = 5/5) were fitted with a tracheal balloon and a carotid artery cannula. Three to 5 days later, the tracheal balloon was inflated for 15 s. Blood samples (0.2 mL each) were collected just before and during the final third of the apnea. The experiment was repeated with an interval of at least one day. PCO_2_, PaO_2_, SaO_2_, and pH were measured using an I-STAT with an EG7+ cartridge (Abbott, New Jersey, USA).

### Data analysis

Arterial and thoracic pressure and ECGs were recorded at 1000 Hz with a PowerLab 8/30 (AD Instruments). Data were analyzed using LabChart 7.1 software (AD Instruments). Heart rate was derived from the ECG. Respiratory effort was calculated for each respiratory cycle as the thoracic pressure swing.

The results are presented as the means ± SEM. Average responses were calculated from the difference between the pre-apnea value and the average level during the 14 s apnea period. Apnea-induced responses before and after treatment were compared by two-way ANOVA with repeated measurements. Significant ANOVAs were followed by Bonferroni’s test (PASW Statistics 18, IBM, Illinois, USA). P-values of <0.05 were considered significant.

## Results

### Behavioral responses to obstructive apnea

During the first 10 s of apnea, respiratory effort increased, but locomotion was rare. During the final 5 s of apnea some locomotion was common, but escape attempts were rare. Rats typically took 2 or 3 deep breaths after the end of apnea, and breathing returned to normal. Behavioral responses were more intense in SHR than in WKY. Behavioral responses induced by apnea were much less intense than responses to i.v. injection of cyanide. Behavioral responses to apnea were abolished by bilateral ligation of the carotid body artery, and by inhaling 100% oxygen before induction of apnea.

### Cardiovascular responses to apnea are more intense in SHR than in WKY rats

**Figure 1:**
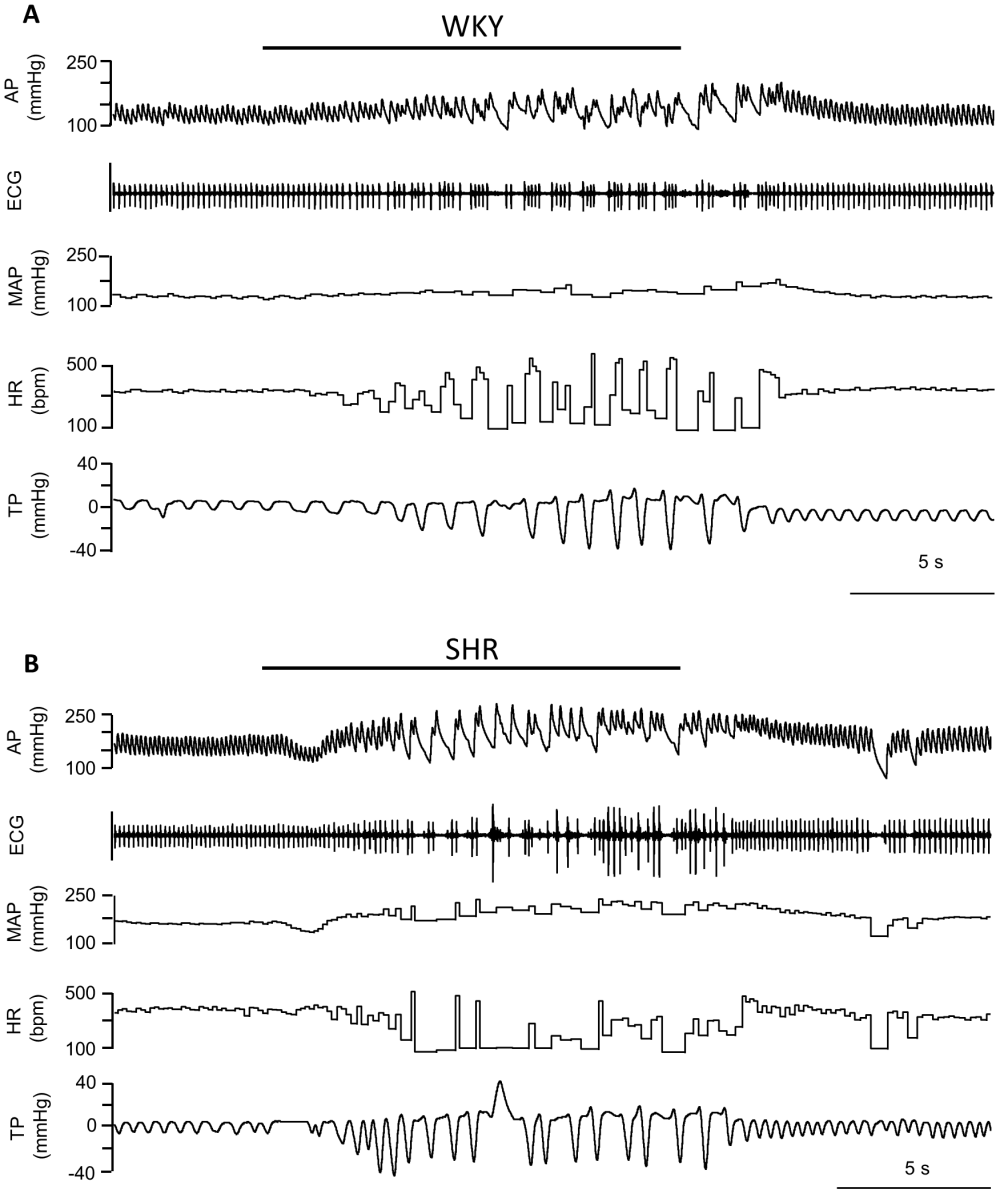
Cardiovascular and respiratory responses induced by obstructive apnea in rats. Representative recordings of arterial pressure (AP), electrocardiogram (ECG), mean arterial pressure (MAP), heart rate (HR) and thoracic pressure (TP) over 15 s of apnea in (A) a normotensive Wistar Kyoto rat (WKY) and (B) a spontaneously hypertensive rat (SHR).

Obstructive apnea produced by the inflation of the tracheal balloon induced a progressive increase in respiratory effort. Arterial blood pressure started to increase after approximately 2 s of apnea, continued to rise during apnea, and further increased during the first few seconds after the end of apnea, before falling back to resting levels. The heart rate fell progressively during apnea. With increasing apnea duration, intense respiratory sinus arrhythmia developed, with a high heart rate during inspiratory effort and only a few beats during expiratory effort. After the end of apnea, the heart rate rapidly increased to resting levels (Fig. 1).

**Figure 2:**
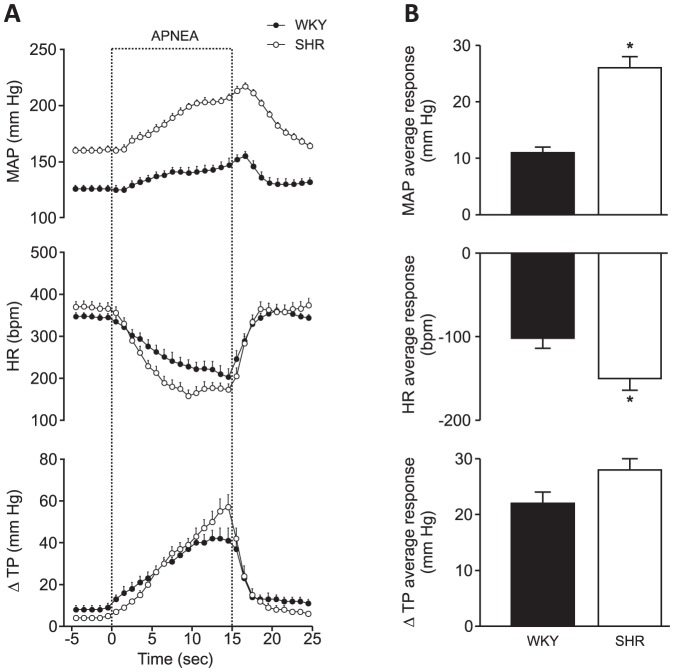
Cardiovascular and respiratory responses induced by obstructive apnea in rats. A. Arterial pressure, heart rate, and breathing effort (measured as thoracic pressure swing) in SHR (n = 14) and WKY (n = 13) over 15 s of apnea. Error bars indicate SEM. MAP  =  mean arterial pressure, HR  =  heart rate, TP  =  thoracic pressure. B. Average change during the apnea period. * indicates a difference between strains (Bonferroni test, p<0.05).

The responses induced by apnea were more intense in SHR than in WKY rats (Fig. 2). The hypertension at the end of apnea was 57±3 mmHg in SHR and 28±3 mmHg in WKY (mean ± SEM, n = 14/13). The heart rate fell by 209±19 bpm in SHR and by 155±16 bpm in WKY. Respiratory effort (measured as the thoracic pressure swing during the respiratory cycle) was 53±6 mmHg in SHR and 34±5 mmHg in WKY. The average pressor and heart rate responses to apnea were higher in SHR than in WKY, whereas increases in respiratory effort were similar in the two strains (Fig. 2B).

### Changes in blood gases during apnea are similar in SHR and WKY strains

**Figure 3:**
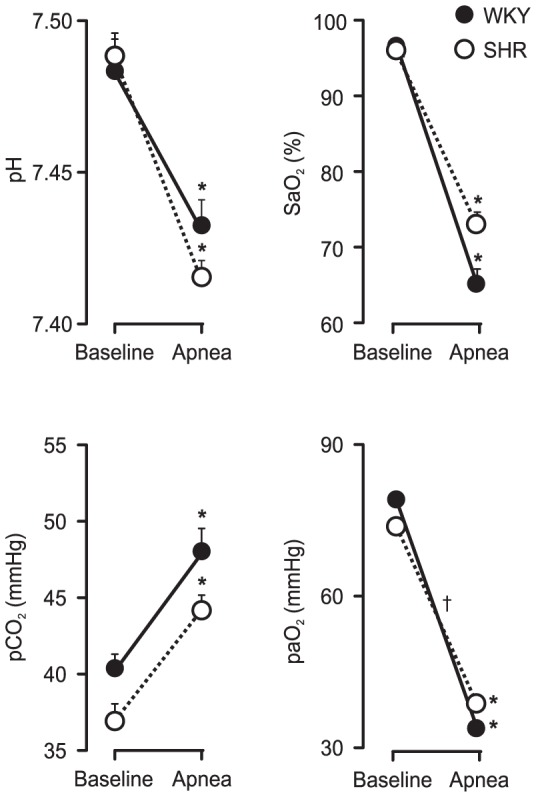
Changes in blood gases during apnea. Arterial blood gases before and after 10 to 15 seconds of apnea in WKY (n = 5) and SHR (n = 5). Error bars indicate SEM. * indicates a difference from the pre-apnea level. † indicates a difference between strains (Bonferroni, p<0.05).

Arterial blood samples were collected before apnea and after 10 – 15 s of apnea in SHR and WKY. As expected, apnea reduced pH, paO_2_ and SaO_2_ and increased pCO_2_ in both strains (Fig. 3). Apnea-induced changes in pH, PCO_2_, and PaO_2_ were similar in SHR and WKY. The changes in SaO_2_ were slightly but significantly larger in WKY.

### Surgical inactivation of carotid chemoreceptors attenuates apnea-induced responses

**Figure 4:**
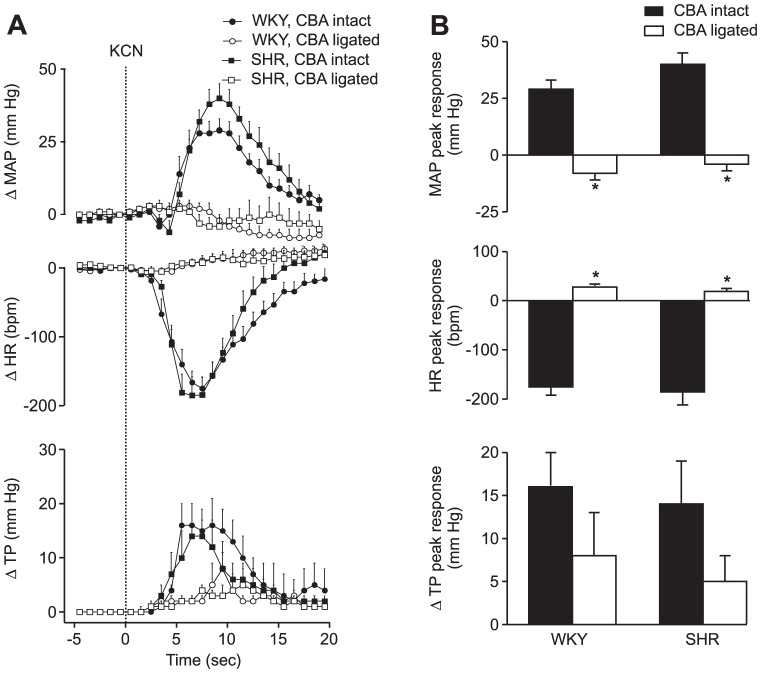
Effect of bilateral ligation of the carotid body arteries on responses induced by cyanide. A. Changes in mean arterial pressure, heart rate and thoracic pressure induced by the i.v. injection of 40 µg potassium cyanide (KCN) in WKY (n = 13) and SHR (n = 14) before and two days after section of the carotid body arteries. Error bars indicate SEM. MAP  =  mean arterial pressure, HR  =  heart rate, TP =  thoracic pressure. B. Peak responses during the KCN test. * indicates a difference from the pre-ligation value (Bonferroni, p<0.05).

To test whether carotid chemoreceptors contribute to the strain differences in apnea-induced cardiorespiratory responses, we compared responses before and after the ligation and section of the carotid body artery (CBA). Chemoreceptor inactivation was verified by the i.v. injection of 40 µg potassium cyanide. Before CBA section, cyanide caused respiratory effort, a profound bradycardia, and a large pressor response, which were similar in the two strains. These responses to cyanide were abolished in all rats 2 days after CBA section (Fig. 4).

**Figure 5:**
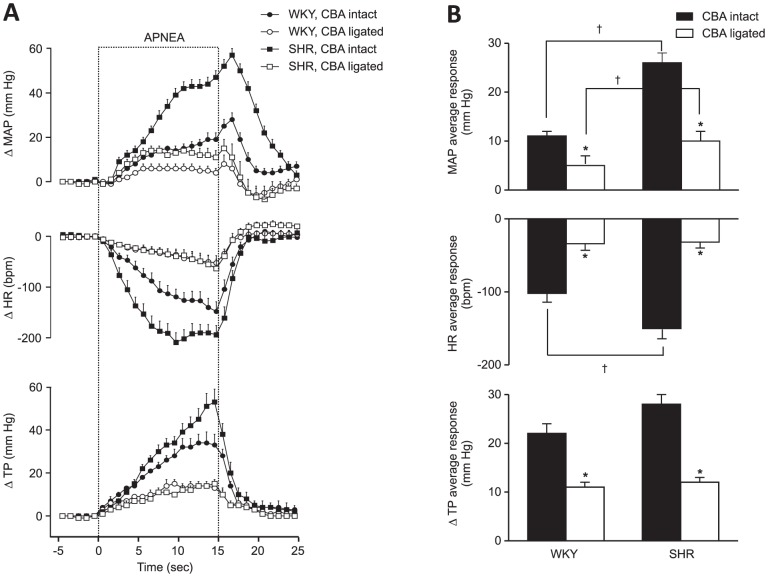
Role of carotid chemoreceptors in responses induced by obstructive apnea. A. Apnea-induced responses in WKY (n = 13) and SHR (n = 14) rats, before and two days after section of the carotid body arteries. Error bars indicate SEM. MAP  =  mean arterial pressure, HR  =  heart rate, TP  =  thoracic pressure. B. Average responses to apnea. * indicates difference from before inactivation; † indicates difference between strains (Bonferroni, p<0.05).

The responses induced by apnea were attenuated after the inactivation of carotid chemoreceptors in both strains. As a consequence, the apnea-induced responses were similar in SHR and WKY rats after CBA ligation (Fig. 5). Carotid chemoreceptor inactivation caused a small reduction in resting arterial blood pressure but did not change heart rate, respiratory effort, and respiratory rate (Table 1).

**Table 1:** Resting arterial pressure, heart rate, thoracic pressure and respiratory rate before and 2 days after ligation of the carotid body arteries (CBA).

	MAP	HR	TPS	RR
	(mm Hg)	(bpm)	(mm Hg)	(cycles/min)
WKY, intact CBA	126±3	346±7	8±2	99±6
WKY, ligated CBA	115±2*	385±10	8±2	92±8
SHR, intact CBA	161±5^†^	365±21	4±0^†^	119±11
SHR, ligated CBA	140±4*^†^	396±17	5±1^†^	116±22

MAP  =  mean arterial pressure, HR  =  heart rate, TPS  =  thoracic pressure swing, RR  =  respiratory rate. Data are the means ± SEM. * difference between intact and inactivated chemoreceptor, ^†^ difference between SHR and WKY (p<0.05, two-way ANOVA with Bonferroni post-hoc test, n = 13/14).

### Inactivation of peripheral chemoreceptors with hyperoxia attenuates apnea-induced responses

**Figure 6:**
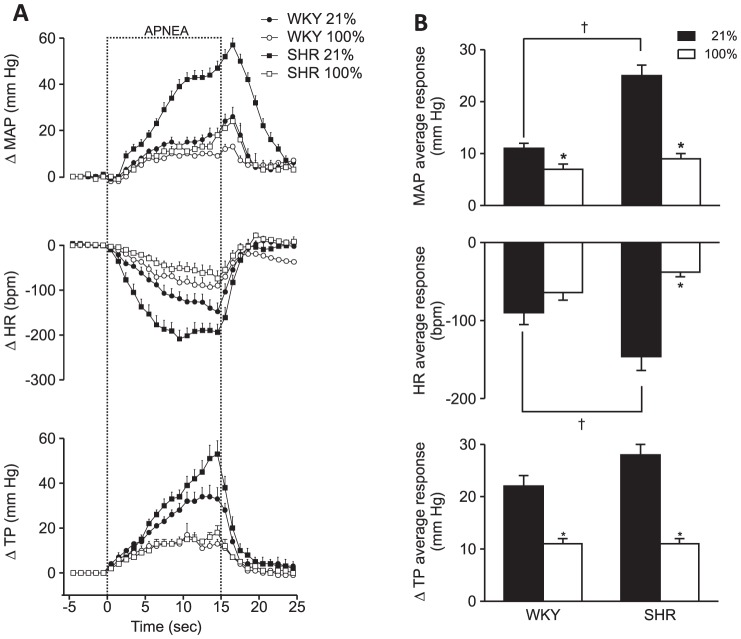
Hyperoxia attenuates apnea-induced responses. A. Changes in mean arterial pressure, heart rate and thoracic pressure in WKY and SHR rats during apnea in normoxia (21% O_2_) and hyperoxia (100% O_2_). Error bars indicate SEM. MAP  =  mean arterial pressure, HR  =  heart rate, TP  =  thoracic pressure. B. Average responses to apnea in WKY (n = 13) and SHR (n = 14) during apnea in normoxia and hyperoxia. Error bars indicate SEM. * indicates a difference from 21%; † indicates a difference between strains (Bonferroni, p<0.05).

In humans, peripheral chemoreceptors can be inactivated by increasing the oxygen content of the inspired air (hyperoxia). The inactivation of peripheral chemoreceptors with hyperoxia in rats reduced apnea-induced hypertension, bradycardia, and respiratory effort in both strains. The reduction in responses induced by hyperoxia was similar to the reduction observed after surgical inactivation. Therefore, the apnea-induced responses were similar in SHR and WKY rats after hyperoxia (Fig. 6).

### In the absence of peripheral chemoreceptors, hyperoxia fails to attenuate apnea-induced responses

**Figure 7:**
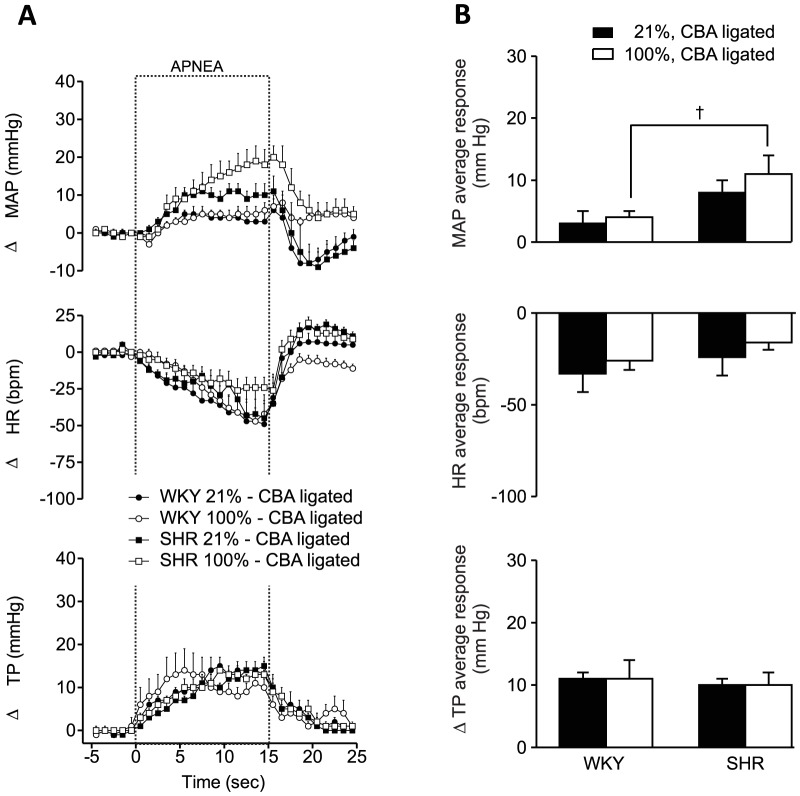
After inactivation of peripheral chemoreceptors, there is no longer a difference between the responses to apnea in 21% and 100% oxygen. A. Effect of hyperoxia on apnea-induced changes in mean arterial pressure, heart rate and thoracic pressure in WKY and SHR rats with surgically inactivated chemoreceptors. Error bars indicate SEM. MAP  =  mean arterial pressure, HR  =  heart rate, TP  =  thoracic pressure. B. Average responses to apnea in WKY (n = 13) and SHR (n = 14) during apnea in normoxia and hyperoxia, both after inactivation of chemoreceptors. † indicates a difference between strains (Bonferroni, p<0.05).

After the surgical inactivation of the carotid chemoreceptors, there was no difference between apneas performed in room air and in 100% oxygen (Fig. 7)

## Discussion

The most interesting new findings of our study were that the cardiovascular responses induced by obstructive apnea were much larger in SHR than in normotensive WKY rats, and that responses to apnea after chemoreceptor inactivation were similar in both strains. Our data show that the stronger responses in SHR are not due to increased hypoxia and hypercapnia during apnea, because changes in blood gases were similar in both strains. We used two methods to inhibit carotid chemoreceptor activation that differ in several ways, but their effects on responses to apnea were identical: both caused a large reduction in apnea-induced cardiovascular responses, and the reduction was larger in SHR than in WKY. As expected, there was no effect of hyperoxia on responses induced by apnea after ligation of the carotid body arteries. Therefore our data suggest that the exaggerated cardiovascular responses to apnea in SHR are due to increased sensitivity of the chemoreflex mechanism.

Several lines of evidence support the idea that chemoreceptor reflexes are enhanced in SHR. The carotid body is larger in SHR than in WKY [11]. The number of glomus cells with strong immunoreactivity for dopamine β-hydroxylase is higher in SHR than in WKY [11]. The glomus cells of SHR respond more strongly to low pH than those of WKY; this response appears to be mediated by the increased expression of the acid-sensing channels ASIC3 and TASK1 [12]. Sympathetic activation upon chemoreceptor stimulation with cyanide was also enhanced in young normotensive SHR compared to WKY [14].

However, apnea-induced responses depend not only on arterial chemoreceptors. Several studies show that sympathetic activity falls immediately after resumption of breathing [21], before the oxygenated blood can reach the carotid chemoreceptors. This fall does not depend on the composition of the air inhaled after apnea: air that is hypercapnic and hypoxic is as effective as room air [22]. Similarly, bradycardia ends immediately when respiration resumes (Fig. 2). Therefore, it seems that the termination of the apnea response does not directly depend on carotid chemoreceptors, but on pulmonary receptors.

It is not completely clear why cardiovascular responses to apnea are clearly increased in SHR and cardiovascular responses to hypoxia are not. In both responses carotid chemoreceptors are important, but signals from other receptors are also involved [22], [23]. As a result, mild or moderate hypoxia induces hyperventilation and increases cardiac output, whereas apnea is met by conservation of body oxygen, which is achieved by reduced perfusion of non-critical tissues and intense bradycardia. Therefore it seems possible that SHR not only have more sensitive carotid chemoreceptors, but that the integration of these various signals is also altered in SHR. In addition, a more intense startle response could contribute to more pronounced responses in SHR, although responses to apnea similar to those seen by us can be observed in decerebrate rats (unpublished observations). The apneas made by us are not very long: when they are made during REM sleep, the rat often fails to wake up [19]. Finally, the much faster changes with apnea may be an advantage because activation of counterregulatory responses is reduced. For example, more intense hyperventilation to hypoxia in SHR [16] could affect the blood gases, reducing cardiovascular changes induced by hypoxia.

Increased cardiovascular responses to chemoreceptor stimulation are also seen in human patients with essential hypertension. Human hypertensive patients have higher resting muscle sympathetic nerve activity, and breathing 100% oxygen reduces muscle sympathetic activity and arterial pressure in hypertensive patients but not in controls [6]. Cardiovascular and respiratory responses to hypoxia [5] and sympathetic activation in response to hypoxia [4] are also more intense in hypertensive subjects. In SHR, denervation of the carotid chemoreceptors reduces hypertension, and inactivation of the chemoreceptors has been proposed for reduction of blood pressure in hypertensive patients [9].

Patients with obstructive sleep apnea also have increased responses to chemoreceptor stimulation. In these patients there is persistent sympathetic activation and hypertension [24], [25], which are reduced by hyperoxia [3]. Several lines of evidence have led to the hypothesis that the repeated stimulation of peripheral and central chemoreceptors associated with intrathoracic pressure oscillations during apnea episodes results in recurrent episodes of sympathetic excitation that, with time, lead to chronic sympathetic activation and arterial hypertension [26]. Intermittent hypoxia increases the responsiveness of carotid bodies to hypoxia [27]. Oxidative stress [28], [29], inflammatory responses [30], and increased endothelin 1 in the carotid bodies [31] may contribute to increased carotid chemoreceptor sensitivity. Repeated hypoxia also changes the neural circuits that control sympathetic activity [32]. Hypertensive patients may be especially sensitive to the deleterious cardiovascular consequences of obstructive sleep apnea because of their increased reactivity to apnea.
